# Cardiac tumors: clinical presentation, diagnosis, treatment, and results

**DOI:** 10.1007/s00423-025-03817-2

**Published:** 2025-07-24

**Authors:** Chonggang Wang, Xiaoyang Li, Lirui Yang, Wenbin Zhang

**Affiliations:** 1 Department of Cardiovascular Surgery, Guizhou Provincial People’s Hospital, No. 52, Zhongshan East Road, Guiyang City, 550002 Guizhou Province China; 2 Department of Oncology, The Affiliated Hospital of GuiZhou Medical University, Guiyang City, Guizhou China; 3 Department of Cardiac Function, Guizhou Provincial People’s Hospital, Guiyang City, Guizhou Province China

**Keywords:** Cardiac tumors, Primary cardiac tumors, Metastatic cardiac tumors, Multimodal diagnosis, Tumor management, Cardiac oncology

## Abstract

Cardiac tumors are a heterogeneous group of diseases that include primary and metastatic tumors. Among primary tumors, benign tumors account for the majority, and the incidence of malignant tumors is low. In contrast, the incidence of metastatic tumors is significantly higher than that of primary tumors. The clinical features of cardiac tumors are diverse, and symptoms vary depending on the tumor type. Therefore, the diagnosis method of cardiac tumors must adopt multi-modal detection methods to ensure the accuracy of diagnosis. Treatment of cardiac tumors mainly involves surgical resection of the primary tumor to ensure complete resection. For metastatic tumors, it is crucial to consider the primary tumor when surgically resecting metastases. Depending on the characteristics of the tumor, appropriate radiation therapy or chemotherapy can improve quality of life and extend survival.

## Introduction

Cardiac mass, while not commonly encountered in clinical practice, is a significant aspect of cardiac disease. Its diagnosis, treatment, and management are therefore crucial. Cardiac mass encompass a variety of lesions, which can be categorized into neoplastic and non-neoplastic types. Neoplastic lesions further include both primary and secondary forms. However, an over-reliance on imaging characteristics for diagnosis complicates their treatment and management. Both benign and malignant tumors, depending on their location and size within the heart, can lead to hemodynamic disorders or arrhythmias. Consequently, gaining a deeper understanding of cardiac tumors is essential.

## Epidemiology

Cardiac tumors can be categorized into primary cardiac tumors, which are further divided into benign and malignant types, or metastases tumors from other organs [1]. The incidence of primary cardiac tumors is exceedingly rare, with a global occurrence rate of 1.38 cases per 100,000 inhabitants annually [2]. According to the latest classification of cardiac tumors proposed by the 5th edition of *WHO Classification of Thoracic Tumours* in 2021, primary cardiac tumors are categorized into benign and malignant types, with the majority being benign, the most common type of this lesion is Papillary fibroelastoma, followed by myxoma and fibroma. Cardiac malignant tumors are exceedingly rare among primary cardiac tumors. Among these, cardiac angiosarcoma (AS), and cardiac leiomyosarcoma (LMS) are the most prevalent, collectively accounting for approximately three-quarters of all cardiac sarcomas [3] (Table 1).

In comparison to primary cardiac tumors, the incidence of metastatic cardiac tumors is significantly higher, being 20 to 40 times more common. Among these, melanoma is the most likely to involve the heart, along with chest tumors such as those arising from lung cancer, breast cancer, and esophageal cancer, which also have a high probability of metastasizing to the heart [4].

**Table 1:** Cardiac tumors

Benign primary cardiac tumors	Malignant primary cardiac tumors	Metastases tumors
Papillary fibroelastoma	Angiosarcoma	lung
Myxoma	Leiomyosarcoma	Hematologic
Fibroma	Pleomorphic sarcoma	Breast
Rhabdomyoma	Neoplasm, metastatic Hematolymphoid tumors	Esophageal
Adult cellular rhabdomyoma	Diffuse large B-cell lymphoma	Skin
Lipoma	Fibrin-associated diffuse large B-cell lymphoma	Stomach
Lipomatous hypertrophy of the atrial septum		Renal cell
Lipomatous hamartoma of atrioventricular valve		Hepatocellular
Hamartoma of mature cardiac myocytes		Sarcoma
Mesenchymal cardiac hamartoma		
Hemangioma		
Venous hemangioma		
Capillary hemangioma		
Arteriovenous hemangioma		
Cavernous hemangioma		
Conduction system hamartoma		
Cystic tumor of atrioventricular node		

## Clinical presentation

The clinical manifestations of cardiac tumors are diverse, ranging from the absence of symptoms detected during physical examination to manifestations such as chest tightness, chest pain, and even sudden cardiac death. It primarily depends on their size, location, proximity to adjacent cardiac structures, and degree of invasiveness.

Most benign tumors do not present with clinical symptoms. In certain common tumors, such as myxoma, the clinical manifestations are typically closely linked to both the size and location of the tumor. Additionally, the symptoms are often associated with obstruction of the atrioventricular valve. When a tumor is located in the left heart system, it may lead to distal vascular embolism, which can result in cerebral infarction, renal and splenic infarction, mesenteric ischemia, and distal limb ischemia. Conversely, if the tumor is situated in the right heart system, it may present as pulmonary embolism. Moreover, if the tumor is of considerable size, it may result in congestion within the cardiac systemic circulation or pulmonary circulation. Additionally, it can obstruct the cardiac outflow tract, potentially leading to acute sudden cardiac death.

Some patients may present with non-specific symptoms, including fatigue, cough, joint pain, and muscle pain. Laboratory results may reveal increased erythrocyte sedimentation rate, anemia, and elevated C-reactive protein (CRP) levels. Notably, dyspnea may worsen even when the patient is lying on their left side [5].

Patients with cardiac malignancies typically present with clinical symptoms at a relatively late stage, which often correlates with the extent of tumor invasion. These symptoms may include rapid weight loss, dyspnea, atypical or pleuritic chest pain, syncope, presyncope, and increased fatigue [6].

## Diagnosis

The diagnosis of cardiac tumors primarily relies on multi-modal non-invasive imaging technologies, which can provide critical information regarding the size, shape, and anatomical location of the tumor. These imaging techniques can also assess whether there is obstruction of blood flow, involvement of the valves, invasion of surrounding tissue, presence of pericardial effusion, and the status of cardiac function. Among these methods, Echocardiography (ECHO), Computed Tomography (CT), and Cardiac Magnetic Resonance (CMR) are the most commonly used diagnostic techniques.

### Echocardiography

Transthoracic Echocardiography (TTE) is the primary method for the preliminary diagnosis of cardiac tumors. Its key advantages include cost-effectiveness, convenience, widespread availability, diagnostic sensitivity, and high spatial and temporal resolution, which facilitate the detection of relatively small and mobile masses. Additionally, Doppler imaging can be employed to assess the status of intracardiac blood flow, aiding in the determination of potential obstruction or valve involvement [7].

Transesophageal Echocardiography (TEE) is more accurate for evaluating masses located on the left side of the heart. It effectively assesses the size and location of these masses, as well as determining the presence of any blood flow obstruction. Additionally, it evaluates valve function and measures cardiac chamber volume reduction following mass resection during surgery.

However, echocardiography has notable disadvantages, including its high dependence on the operator’s skill level and its limited acoustic window. These factors particularly pose significant challenges for obese patients and those with chronic lung diseases.

### Computed tomography

As diagnostic technology advances, cardiac computed tomography has become increasingly utilized for the evaluation of cardiac masses, particularly when echocardiography is inconclusive or contraindicated. CT offers high spatial resolution cross-sectional imaging of the heart and its surrounding structures, establishing it as the diagnostic modality of choice for suspected cardiac metastases or primary pericardial tumors.

Multi-detector computed tomography (MDCT) with electrocardiogram (ECG) gating is a rapid imaging technique that yields high-quality cardiac images due to its superior spatial and temporal resolution. Images can be acquired at any or all stages of the cardiac cycle and reconstructed in any desired plane. However, its disadvantages include the use of ionizing radiation and iodinated contrast agents, as well as lower soft tissue contrast resolution and temporal resolution when compared to echocardiography and CMR [8].

### Cardiac magnetic resonance

Cardiac magnetic resonance generates images of the heart by applying a magnetic field to align the weak intrinsic magnetic moments of hydrogen protons within the body. CMR imaging offers multiplanar capabilities, a wide field of view, and high spatial and temporal resolution, along with excellent intrinsic soft tissue contrast, all without the use of ionizing radiation or iodinated contrast agents. Specific imaging sequences can be employed to emphasize different tissue characteristics, and the application of intravenous contrast agents can enhance the understanding of the internal composition of the mass [9]. In comparison to echocardiography and CT, CMR does not require radiation exposure or nephrotoxic contrast agents, and it is not subject to acoustic window restrictions. Most importantly, CMR offers superior tissue characterization. However, due to the relatively high costs, extended acquisition times, restricted environments, and the necessity for patient cooperation, CMR may not be appropriate for all patients. Moreover, due to its lower time resolution compared to echocardiography, valve function cannot be assessed in patients whose heart valves are affected by tumors. Additionally, there are contraindications for patients with claustrophobia or those who have older cardiac devices implanted [10] (Table 2).

**Table 2:** Comparison of the advantages and disadvantages of different imaging diagnostic technologies

Modality	Multiplanar Capability	Soft-Tissue Contrast	Temporal Resolution	Windows limited	Price	Time	Radiation
ECHO	+++	+	++++	+	+	+	-
CT	++	++	+	-	++	++	+
CMR	++++	++++	+++	-	+++	++++	-

## Therapy

The treatment of cardiac tumors is contingent upon several factors, including the type of tumor, the certainty of diagnosis, the clinical symptoms presented, and the presence of comorbidities or metastatic spread.

For benign cardiac tumors located in the left heart system, the risk of embolism is a significant concern, and surgical resection is routinely recommended, even for small tumors. Conversely, for tumors situated in the right heart system, if the patient exhibits no obvious symptoms and does not have a patent foramen ovale or atrial septal defect, strict regular follow-up may be sufficient. Surgical resection can then be considered if deemed necessary. All symptomatic benign cardiac tumors necessitate surgical resection [11].

For malignant cardiac tumors with non-infiltrating margins, radical surgery is the preferred approach to prevent recurrence and improve survival. However, in certain cases of the infiltrative growth of the tumor, along with anatomical abnormalities or its proximity to critical cardiac structures, achieving complete R0 resection is challenging, and R1/R2 resections are associated with early recurrence and lower survival rates. Therefore, multimodality treatment, including pre- and/or postoperative chemotherapy and radiotherapy, is widely accepted, even in localized, non-metastatic cases [12, 13]. If the tumor does not metastasize, transplantation has been considered for select cases, but its long-term outcomes and precise indications remain unclear [14]. Consequently, the necessity of cardiac transplantation remains a subject of debate.

For secondary cardiac tumors, a comprehensive assessment must consider the patient’s condition, including the location and malignancy of the primary tumor. If metastasis leads to hemodynamic disorders, palliative surgical treatment should be performed based on the patient’s condition to alleviate symptoms [15].

## Benign tumors

Among primary cardiac tumors, the majority are benign, with papillary fibroelastoma (PF) being the most common type, followed by myxomas, fibromas [3].

### Papillary fibroelastoma

Fibroelastoma, also known as papillary fibroelastoma, is a benign tumor that typically arises from the endocardium, most commonly located on the surface of heart valves. This tumor accounts for approximately three-quarters of all tumors found on heart valves [16]. PF can occur across all age groups, from newborns to elderly period, with an average onset age of approximately 60 years. Additionally, no significant gender predisposition has been identified [17].

PF can be described as round, oval, or irregular in shape, typically exhibiting a leaf-like appearance. It features multiple long and narrow papillary branches and has a soft, brittle texture, often resembling an anemone [18]. Histologically, the PF is characterized by a central avascular collagen core composed of dense connective tissue, which is encircled by a layer of loose connective tissue. This outer layer contains proliferating endocardial cells that possess organelles and cysts [19]. The pathological origin of PF remains unclear, with several etiological hypotheses proposed. These include hamartomas, true tumors, endocardial proliferative responses to genetic stimuli or external factors, and organized thrombi [16].

#### Clinical symptoms

Typically, due to the small size of the PF and its minimal impact on hemodynamics, there are often no specific symptoms associated with it. However, PF may present with a range of nonspecific signs and symptoms, including peripheral vascular emboli and impacts on valve function [20–22]. Systemic symptoms such as fever, dyspnea, palpitations, and chest discomfort may also manifest [23–25]. Furthermore, there have been reports of unexpected associations with left ventricular apical ballooning syndrome, subacute bacterial endocarditis, antiphospholipid antibody syndrome, thyroid dysfunction, and thrombocytopenia [26, 27].

#### Diagnosis

Due to the non-specific symptoms of the clinical features associated with PF, it is often identified during surgical procedures or autopsies, among various diagnostic modalities, echocardiography remains the most accessible, convenient, and effective diagnostic method, the PF can be accurately detected by echocardiography and even the small mobile masses attached to the valve by a short pedicle [28]. In a case-control study, the sensitivity and specificity of TTE for detecting PF ≥ 0.2 cm were 88.9% and 87.8%, respectively, with an overall accuracy of 88.4%. When the PF is < 0.2 cm, the overall sensitivity of TTE is 61.9%, while the sensitivity of TEE is 76.6% [18].

#### Treatment

For the treatment of PF, the treatment plan must be tailored to the patient’s specific condition. If the patient has a history of embolism or if the tumor is mobile and located within the left heart system, surgical intervention should be considered [29]. For patients with contraindications to surgery, anticoagulant therapy may be considered to prevent embolization. Once the conditions for surgery are met, the surgical intervention should be performed at the earliest opportunity [17].

For asymptomatic patients, the necessity of surgical treatment hinges on individualized treatment plans tailored to specific circumstances. Surgical intervention is prioritized if the tumor is mobile and poses a particular risk of embolism. In cases where the mobile tumor ≥ 1 cm, which heightens the risk of embolism or sudden cardiac death, aggressive surgical treatment becomes essential [18]. Conversely, if the tumor measures < 1 cm and the patient exhibits no discernible symptoms, a strategy of regular observation and follow-up, along with consistent echocardiographic monitoring, is recommended until pertinent surgical indications arise [30]. However, a unified consensus has not yet been achieved, and many aspects remain controversial.

### Myxoma

Cardiac myxoma is among the most prevalent primary benign cardiac tumors, with an incidence rate exceeding 50% [31]. 90% patients develop the disease between the ages of 30 and 60, with the average age at diagnosis being 50 years old. Furthermore, the incidence rate in women is 1 to 3 times higher than that in men [32]. However, myxoma is relatively uncommon in children [33]. Most myxomas originate from the interatrial septum adjacent to the atrial fossa ovalis; however, they may occasionally arise from the posterior chamber wall, anterior chamber wall, or heart valves, though such occurrences are extremely rare. And approximately three-quarters of myxomas originate in the left atrium, but also one-third of these patients are associated with thrombus [34].

In addition, there is a special dominant chromosomal genetic disease, carney complex, is a rare multiple neoplasia and lentiginosis syndrome, which is characterized by multiple endocrine and non-endocrine tumors, including cardiac myxoma [35]. Notably, this cardiac myxomas can occur in multicentric and have a high tendency to recur after surgical resection, with a reported recurrence rate of 44% [36]. Recent studies have identified that the Carney complex may result from defects in the PPKAR1α gene located on chromosome 17q24 [37].

Myxomas are typically polypoid, exhibiting a round or oval shape, and possess a smooth or slightly lobulated surface. They are generally attached to the interatrial septum via a pedicle. The mobility of the tumor varies according to its collagen content, the degree of attachment, and the length of the pedicle [34].

#### Clinical symptoms

Similar to other cardiac masses, the characteristics of myxomas are influenced by their location, size, and mobility. In many cases, cardiac myxoma may be asymptomatic, particularly when the tumor is small and does not exert a significant hemodynamic impact. Physical symptoms of cardiac myxoma may encompass fever, palpitations, anorexia, joint pain, and weight loss. If the myxoma is large and located in the left heart system, it primarily results in symptoms associated with mitral valve obstruction or regurgitation, which can lead to left-sided heart failure and secondary pulmonary hypertension. Key clinical manifestations may include exertional dyspnea, orthopnea, and pulmonary edema. A right-sided myxoma presents with cardiovascular signs that resemble tricuspid stenosis and right heart failure. Common symptoms include dyspnea on exertion, peripheral edema, hepatomegaly, and ascites [38]. In addition, thrombus formation on the surface of a tumor can result in systemic embolism, which may present as systemic infection, transient ischemic attack, stroke, hemiplegia, visual impairment, chest discomfort, and dyspnea [39, 40].

#### Diagnosis

In most cases, myxoma can be definitively diagnosed through echocardiography due to its characteristic location and the nature of its pedicle, as well as its impact on hemodynamic and valvular abnormalities. According to reports, the detection rate of cardiac myxoma using transthoracic echocardiography (TTE) and transesophageal echocardiography (TEE) is reported to be between 95% and 100% [41]. In more complex cases, such as right atrial myxoma, MRI and CT imaging may provide additional insights [38, 42]. Additionally, for small masses, a course of oral anticoagulants can help distinguish between myxoma and thrombus [43, 44].

#### Treatment

Upon the discovery of a cardiac myxoma and the diagnosis has been confirmed through imaging, surgical resection is typically the first course of action, as it offers immediate relief from associated complications and provides a more durable solution [1]. And this treatment method has been demonstrated to be the most direct and effective, achieving a 5-year survival rate of 98.4 ± 1.6% and a 10-year survival rate of 96.0 ± 2.8% [42].

Currently, several treatments are available for patients with cardiac myxoma. The most important method and the gold standard of treatment remains conventional thoracotomy or minimally invasive surgery. This procedure must be performed by an experienced surgeon and requires the complete removal of the tumor and the entire base [45]. The most emerging treatment modality currently involves robotic-assisted surgery, which is regarded as an advanced approach for the management of cardiac myxomas [46].

**Fig. 1:**
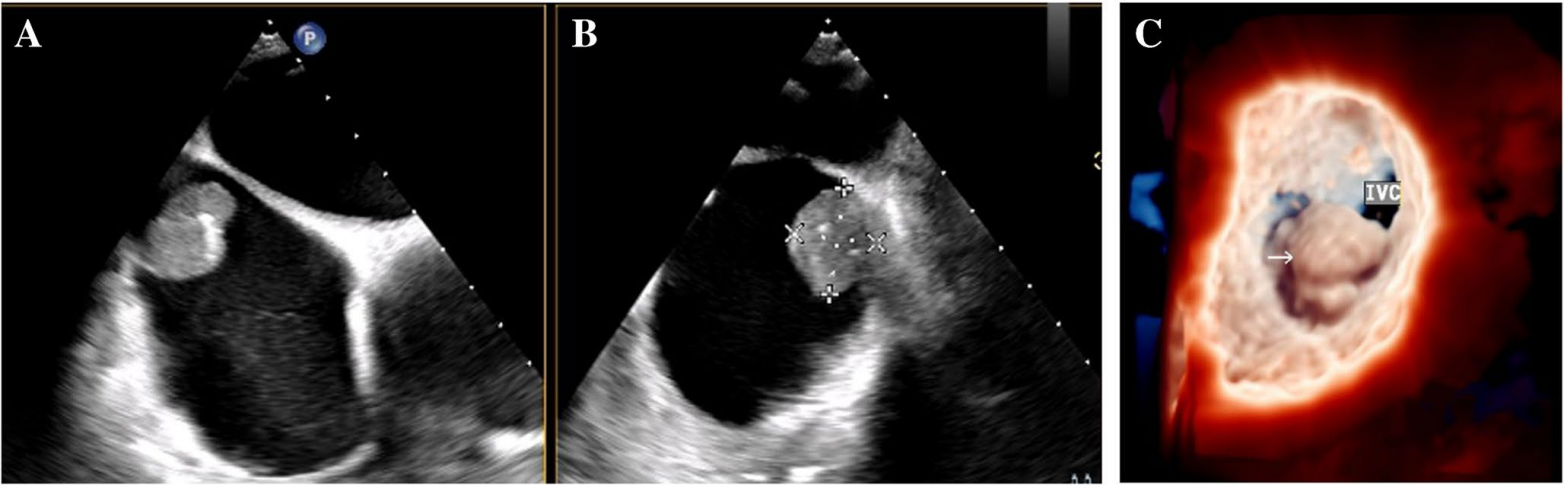
Transthoracic echocardiography (**A** and **B**) and transesophageal echocardiography 3D imaging (**C**) of right atrial myxoma

To prevent the recurrence of myxoma and complications such as arrhythmia following surgery, regular postoperative follow-up is essential, generally, patients who have undergone surgical resection of an myxoma should be followed up within one year post-surgery, followed by evaluations every five years thereafter [45, 47]. Typically, postoperative follow-up can be conducted through clinical examination, TTE or TEE [48]. Familial cardiac myxoma can be evaluated using CT or MRI for further auxiliary examination, which may provide clearer insights and facilitate the early detection of potential recurrence [49] (Fig. 1).

### Fibroma

Cardiac fibromas are distinctive connective tissue tumors that are typically solitary and invariably located in the ventricles. These tumors are usually well-circumscribed, often exhibit central calcification, and are devoid of cystic changes, necrosis, or hemorrhage. They represent the most common type of heart tumor in children, with one-third of affected patients being younger than 1 year old and can range in size from 1 to 10 centimeters [50, 51]. Fibroids can infiltrate the ventricular muscle, leading to refractory congestive heart failure. Additionally, they may extend into the ventricular conduction system, resulting in ventricular arrhythmias, and these condition that is not uncommon [51, 52].

For the treatment of fibromas, surgical resection is undoubtedly the most effective option for patients with symptomatic, resectable tumors. However, if a significant portion of the functioning heart is compromised by the tumor, it may lead to uncontrollable heart failure or arrhythmias. In such cases, a heart transplant may be the only viable option to consider [53, 54].

## Malignant tumors

Malignant primary tumors are exceedingly rare, with only approximately 25% of primary cardiac tumors classified as malignant. Among these primary malignant tumors, the vast majority (95%) are sarcomas, which include angiosarcoma, rhabdomyosarcoma, leiomyosarcoma, liposarcoma, osteosarcoma, fibrosarcoma, and malignant fibrous histiocytoma. The remaining 5% consist of primary cardiac lymphomas and mesothelioma [55, 56].

### Sarcomas

Sarcoma is diagnosed in young adults aged 30 to 50 years, with a mean age of 46 years, and it affects both sexes in nearly equal proportions. This condition is characterized by a dismal and rapidly progressive clinical course, resulting in a median survival of only one year [57, 58]. Recent research statistics indicate that the incidence rates of angiosarcoma and leiomyosarcoma are the highest among all types of sarcomas [3].

### Angiosarcoma

Angiosarcoma represents the most prevalent histological subtype of cardiac sarcoma. It is more frequently observed in middle-aged men, with a male-to-female ratio of approximately 2:1. In comparison to other malignant tumors affecting the heart, angiosarcoma is associated with the poorest prognosis [59]. Approximately 75% of angiosarcomas are found in the right heart, particularly the right atrium. These tumors may also invade adjacent structures, including the pericardium, inferior vena cava, and tricuspid valve.

By the time the disease is diagnosed, approximately 80% of patients have already developed metastatic tumors [60]. The most frequent sites of metastasis are the lungs and bones; however, the spleen and liver can also be involved. Occasional metastases to the brain may occur. The clinical features of angiosarcoma often encompass chest pain, dyspnea, cardiac arrhythmias, and malignant pericardial or pleural effusion, and some patients may exhibit symptoms of heart failure, pulmonary hemorrhage, or metastasis to other organs caused by the tumor [61–65].

Surgical resection is considered the treatment of choice for angiosarcoma. However, due to the tendency for these tumors to exhibit local invasion and frequent metastasis at the time of diagnosis, achieving complete resection is often unattainable [65]. However, surgery alone is insufficient to prevent the recurrence of primary tumors or the occurrence of distant metastasis. Even when a significant number of patients undergo complete tumor resection, the possibility of recurrence remains. Consequently, multiple systemic treatment, encompasses both preoperative and postoperative chemotherapy and radiotherapy, becomes even more critical, which can prolong survival time effectively [66–68].

### Leiomyosarcoma

Leiomyosarcoma is a type of soft tissue sarcoma characterized by cells exhibiting distinct smooth muscle traits [59]. This is a rare, highly aggressive malignant tumor, it constitutes merely 8-9% of the incidence of primary malignant cardiac tumors [32].

These tumors are most frequently located in the left atrium, and present as sessile masses, which may exhibit a mucoid appearance. Typically, the age of onset occurs within the 40 to 50-year age group. And the primary clinical symptoms may include dyspnea, chest pain, and a nonproductive cough. However, instances of right heart failure, valvular stenosis, arrhythmias, hemopericardium, and sudden death have been reported on occasion [69].

Due to the extremely low incidence of this tumor, the exact treatment remains uncertain, and to date, there has been no consensus on the optimal treatment strategy. Due to the progressive nature of malignant tumors and the subsequent development of related complications, radical surgery appears to be the preferred strategy, offering greater hope for alleviating the disease and extending life expectancy [70]. However, even when surgical treatment is undertaken, the prognosis remains bleak, as patients typically seek treatment only after experiencing various complications, by which time tumor metastasis has often already occurred. Even with radical surgical intervention, recurrence and metastasis are common, resulting in a generally low average survival rate, typically less than one year [71].

## Metastatic cardiac tumors

In comparison to primary cardiac tumors, metastatic cardiac tumors are significantly more prevalent, with an incidence that is 20 to 30 times greater than that of primary cardiac tumors [72].

Primary tumors that metastasize to the heart can be categorized into three types based on their incidence rates: (1) Rare primary tumors with high metastasis rates, such as malignant melanoma, which can exceed a metastasis rate of 50% [73]. (2) Common tumors with moderate cardiac metastasis rates, which account for the largest number of cardiac metastases, including lung cancer, gastric cancer, liver cancer, ovarian cancer, and other malignant tumors that can disseminate through the circulatory system [60, 74, 75]. (3) Common tumors that infrequently metastasize to the heart, such as cervical cancer [76, 77]. Then the primary malignancies can metastasize to the heart via four pathways: direct spread, hematogenous spread, lymphatic spread, and intraluminal extension within the vasculature, such as extension of the superior and inferior vena cava as well as the pulmonary veins [78]. And the area of the heart affected by the tumor may be either localized or diffuse.

The clinical manifestations of cardiac metastatic tumors primarily depend on the site of metastasis, as these tumors can localize in any region of the heart. However, the pericardium is the most commonly affected area, followed by the myocardium and endocardium [74] with occasional involvement of the coronary arteries. Additionally, obstruction may occur due to expansion in the vena cava.

**Fig. 2:**
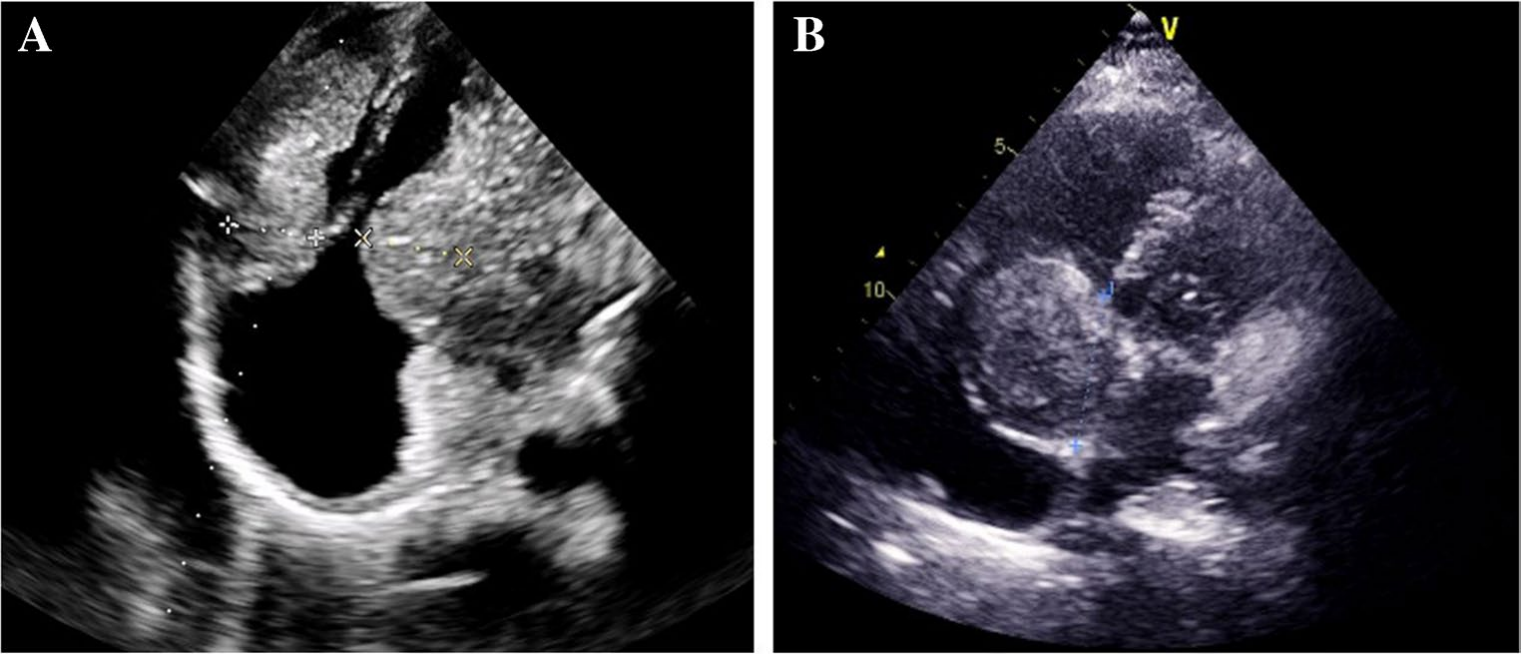
Metastatic tumor invading the left and right ventricles (**A**) and right ventricles (**B**)

The management of metastatic cardiac tumors primarily depends on the type of the primary tumor. Surgical resection of metastatic tumors remains the primary option for alleviating symptoms. Additionally, in cases complicated by recurrent pericardial effusion, the drainage of the pericardial window is an essential procedure. And depending on the type of primary tumor, chemotherapy or radiotherapy may be essential treatment modalities. However, it is crucial to consider the cardiotoxicity associated with chemotherapy agents and the potential complications that may arise [79] (Fig. 2).

## Conclusions

In general, cardiac tumors represent a heterogeneous group of diseases, encompassing both primary (benign and malignant) and metastatic tumors. Among primary tumors, benign tumors constitute the majority, while the incidence of malignant tumors remains relatively low. In contrast, metastatic tumors exhibit a significantly higher incidence rate compared to primary tumors. The clinical characteristics of cardiac tumors vary, leading to different symptoms based on the specific type of tumor. Consequently, the diagnostic approach for cardiac tumors necessitates the use of multi-modal detection methods to ensure an accurate diagnosis. Among these methods, echocardiography is the primary diagnostic tool, with cardiac CT and cardiac CMR serving as important adjuncts for further evaluation. The treatment of cardiac tumors primarily involves the surgical resection of primary tumors to ensure complete removal and to prevent the occurrence of related complications. In the case of metastatic tumors, it is essential to consider the primary tumor during the surgical resection of the metastatic lesion. Based on the characteristics of the tumor, appropriate radiotherapy or chemotherapy may be administered to enhance quality of life and prolong survival.

Overall, cardio-oncology has emerged as a well-established subdiscipline. Cardiac tumors represent a distinct category of disease that necessitates a multidisciplinary collaborative approach to treatment.

## Data Availability

No datasets were generated or analysed during the current study.
